# A high density SLAF-seq SNP genetic map and QTL for seed size, oil and protein content in upland cotton

**DOI:** 10.1186/s12864-019-5819-6

**Published:** 2019-07-22

**Authors:** Wenwen Wang, Ying Sun, Peng Yang, Xiaoyan Cai, Le Yang, Junrui Ma, Yuncan Ou, Tianpeng Liu, Iftikhar Ali, Dajun Liu, Jian Zhang, Zhonghua Teng, Kai Guo, Dexin Liu, Fang Liu, Zhengsheng Zhang

**Affiliations:** 1 College of Agronomy and Biotechnology, Southwest University, Chongqing, 400716 China; 2 State Key Laboratory of Cotton Biology/Institute of Cotton Research, Chinese Academy of Agricultural Sciences, Anyang, 455000 China

**Keywords:** Upland cotton, SLAF-seq, Seed size, Oil, Protein, QTL

## Abstract

**Background:**

Cotton is a leading natural fiber crop. Beyond its fiber, cottonseed is a valuable source of plant protein and oil. Due to the much higher value of cotton fiber, there is less consideration of cottonseed quality despite its potential value. Though some QTL controlling cottonseed quality have been identified, few of them that warrant further study are known. Identifying stable QTL controlling seed size, oil and protein content is necessary for improvement of cottonseed quality.

**Results:**

In this study, a recombinant inbred line (RIL) population was developed from a cross between upland cotton cultivars/lines Yumian 1 and M11. Specific locus amplified fragment sequencing (SLAF-seq) technology was used to construct a genetic map that covered 3353.15 cM with an average distance between consecutive markers of 0.48 cM. The seed index, together with kernel size, oil and protein content were further used to identify QTL. In total, 58 QTL associated with six traits were detected, including 13 stable QTL detected in all three environments and 11 in two environments.

**Conclusion:**

A high resolution genetic map including 7033 SNP loci was constructed through specific locus amplified fragment sequencing technology. A total of 13 stable QTL associated with six cottonseed quality traits were detected. These stable QTL have the potential for fine mapping, identifying candidate genes, elaborating molecular mechanisms of cottonseed development, and application in cotton breeding programs.

**Electronic supplementary material:**

The online version of this article (10.1186/s12864-019-5819-6) contains supplementary material, which is available to authorized users.

## Background

As one of the world major economic crops, cotton plays an important role in society. Fiber is the main product of cotton, providing raw materials for the textile industry [1]. In addition to lint (‘fiber’), cottonseed is comprised of kernel, hull and fuzz. Cottonseed kernels are regarded as the best source of vegetable protein after soybean and the fifth most important oil crop after soybean, palm, canola and sunflower [2, 3]. Fiber yield and fiber quality, as well as cottonseed quality traits including seed index, oil percentage and protein percentage are quantitative traits. A previous study had reported the correlation among yield, fiber quality and cottonseed quality traits. Pahlavani et al. [4] reported that oil content was largely affected by seed size. Kothari et al. [5] reported positive relationships for seed oil with fiber strength, uniformity index, and fiber length. Positive correlations were found for seed protein and several agronomic traits whereas negative correlations were found between oil and lint yield along with other agronomic traits. Moreover, cottonseed oil content was also negatively related to seed protein content [6].

The much higher value of cotton fiber made it a primary objective of cotton breeding in the past, which resulted in less consideration of cottonseed quality including oil and protein contents [7]. A recent survey suggested that approximately 5000 QTL had then been identified in cotton [8], which included QTL related to cottonseed quality [7, 9–14]. In addition, QTL associated with seed index and oil content had been identified through GWAS enabled by development of sequencing technology and release of cotton reference genomes [15–18]. However, few stable QTL could be identified for further study.

In past years, simple sequence repeat (SSR) markers had been used to construct many genetic maps in crop research. However, the low polymorphism rate of SSR markers in cotton made it difficult to construct a saturated genetic map, which limited the application of the genetic map in DNA marker assisted selection (MAS). Due to their abundance across the whole genome, single nucleotide polymorphism (SNP) markers became popular in genetic map construction and MAS in recent years [19, 20]. With the rapid development and application of NGS technologies, many complexity reduction approaches have been developed to identify SNPs, such as restriction site-associated DNA sequencing (RAD-Seq) [21] specific locus amplified fragment sequencing (SLAF-seq) [22], and genotyping-by-sequencing (GBS) [23]. Compared with other sequence technologies, SLAF-seq has many merits including: 1) no requirement for a reference genome sequence and polymorphism information; 2) repetitive sequences can be avoided; and 3) a balance between marker density and population size can be maintained by varying the fragment size [22]. In addition, the release of genome sequences of *G. raimondii*, *G. arboreum*, *G. hirsutum* and *G. barbadense* facilitated the application of NGS technology in cotton research [15–18]. Recently, SLAF-seq was applied for genetic map construction, QTL identification and variation analysis in cotton [22, 24]. For example, Ali et al. [22] constructed a high-density genetic map containing 6254 single nucleotide polymorphism markers which covered 3141.72 cM and identified 95 QTL for fiber quality traits. Shen et al. [24] harbored 132,880 SNPs and 6296 InDels between the reference genome (TM-1) and the five tetraploid cotton species, including *G. hirsutum* cv. Emian22, *G. barbadense* acc. 3–79, *G. tomentosum*, *G. mustelinum* and *G. darwinii*. Zhang et al. [25] constructed a genetic map including 5521 high-quality SNP markers by SLAF-seq and detected 18 QTL associated with boll weight.

In this study, a recombinant inbred line (RIL) population of 180 lines was developed from a cross between two upland cotton cultivars/lines, Yumian 1 and M11. Then, SLAF-seq was applied to genotype RILs. The present study aims to construct a high-density genetic map to identify QTL for seed index, cottonseed oil and protein content in upland cotton. The results will facilitate future molecular breeding programs to better exploit the full economic potential of cotton.

## Results

### Phenotypic performance

Descriptive statistics for all traits across three environments were shown in Additional file 5: Figure S1 and Additional file 1: Table S1. Both the skewness and kurtosis values of six traits, including hundred seed weight (HSW, g), hundred kernel weight (HKW, g), ten kernel length (TKL, mm), ten kernel width (TKW, mm), oil (KOC) and protein (KPC) content, were < 1.0 in three environments, which indicated that all traits did not deviate significantly from a normal distribution. Subsequently, correlation analysis across three environments was conducted separately (Additional file 2: Table S2). All traits showed significant correlations with other traits except kernel length, which had normal correlations with KOC and KPC. In addition, KPC showed significant negative correlations with others. The variation among genotypes and environments was highly significant for all test traits, which indicated the influence of each of these factors on cottonseed growth (Additional file 3: Table S3).

### SLAF-seq data analysis and SNP marker development

Restriction fragments ranging from 314 bp to 344 bp were selected for further analysis. These fragments were distributed approximately evenly over the genome (Additional file 6: Figure S2). After sequencing, a total of 452.32 M paired end reads were generated for the two parents and the RIL lines, and 93.81% of these bases were of high quality with Q30 (indicating a 0.1% chance of an error) and average GC content of 38.47%. In total, 60,718,390 reads (26,086,993 for Yumian 1, 29,906,736 for M11 and 4,724,661 for RILs) were obtained. Among these clean reads, the percentages of reads anchored on the reference genome for Yumian 1, M11 and 86 RILs were 99.48, 99.54 and 99.52%, respectively. The percentages of reads properly mapped on the reference genome for Yumian 1, M11 and the RILs were 91.37, 89.8 and 93.62%, respectively (Additional file 4: Table S4). The SLAF number for Yumian 1 was 709,329 with an average sequencing depth of 33.14-fold. For M11, the SLAF number was 718,771 with an average sequencing depth of 38.15. For the RIL lines, 396,418 SLAFs were obtained with average depth of 12.08 (Additional file 4: Table S4). Among these SLAFs, 316,514 SNPs were identified, and 36,161 (11.42% of) SNPs showed polymorphism in the RIL population. Based on the character of the RIL population, only aa × bb polymorphisms were used for further analysis. This type included 21,632 members. After multiple filtering, 7033 SNPs with average sequencing depth of 19.09 were used to construct the genetic map.

### Genetic map construction

**Fig. 1:**
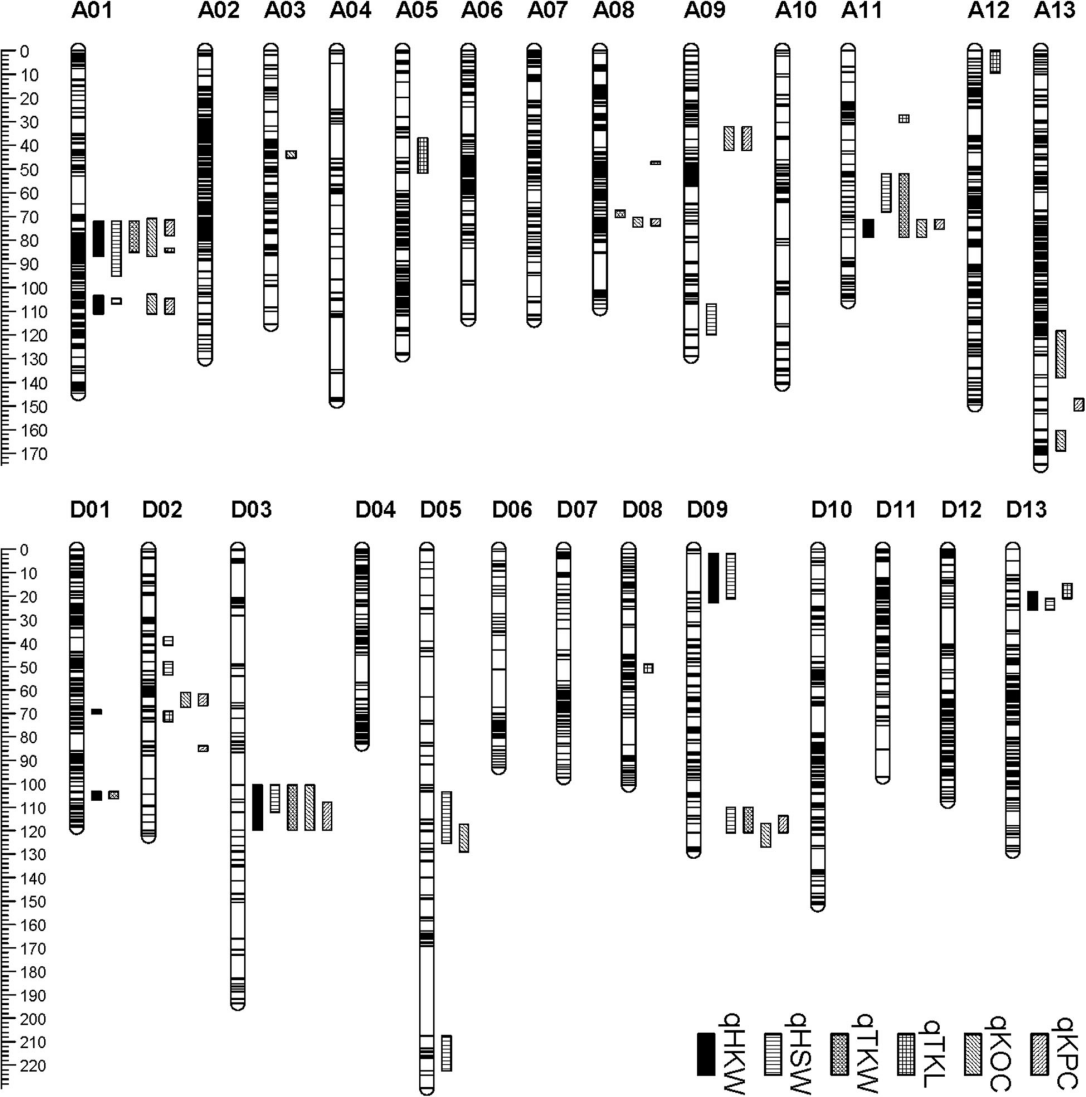
Genetic maps and QTL for cottonseed quality in the Yumian 1 × M11 RIL population

By genetic linkage analysis, a total of 7033 loci were mapped on 26 chromosomes, covering 3353.15 cM with an average distance of 0.48 cM between consecutive markers. Among the 7033 loci, the At genome contained 4295 loci spanning 1701.91 cM at an average of 0.40 cM between adjacent markers, whereas the Dt genome included 2738 loci spanning 1651.24 cM with an average of 0.60 cM between adjacent markers. Chromosome A13 (703 loci) contained the maximum loci, followed by A01 (644) and A02 (613), whereas the fewest were on D06 (109), with an average of 270 loci on each chromosome. The longest chromosome was D05 (229.75 cM), and the shortest was D04 (83.06 cM), with an average chromosome length of 128.97 cM (Fig. 1; Table 1; Additional file 7: Supplement 1).

**Table 1:** Characteristics of the genetic map

Linkage	Total	Total	Average	Gap	Segregation
Group ID	Marker	Distance (cM)	Distance (cM)	< 5 cM(%)	distortion
chrA01	644	144.59	0.22	99.69%	3
chrA02	613	130.01	0.21	99.84%	33
chrA03	189	115.47	0.61	97.34%	31
chrA04	217	169.35	0.78	93.85%	7
chrA05	313	128.33	0.41	98.08%	7
chrA06	180	113.43	0.63	98.32%	1
chrA07	252	113.8	0.45	98.41%	0
chrA08	232	108.79	0.47	98.27%	0
chrA09	235	128.88	0.55	97.86%	6
chrA10	340	247.25	0.73	95.18%	3
chrA11	110	105.76	0.96	96.33%	2
chrA12	267	149.43	0.56	98.87%	32
chrA13	703	175	0.25	99.15%	1
At	4295	1830.09	0.43	98.05%	126
chrD01	266	118.54	0.45	99.25%	30
chrD02	178	122.29	0.69	97.18%	15
chrD03	170	193.56	1.14	93.62%	0
chrD04	150	83.06	0.55	99.33%	0
chrD05	235	229.75	0.98	94.29%	26
chrD06	109	93.05	0.85	96.30%	82
chrD07	223	97.26	0.44	98.65%	0
chrD08	109	100.47	0.92	97.22%	0
chrD09	159	128.66	0.81	97.47%	13
chrD10	415	151.51	0.37	98.79%	24
chrD11	263	97.01	0.37	98.85%	0
chrD12	206	107.39	0.52	98.54%	0
chrD13	255	128.69	0.5	98.43%	61
Dt	2738	1651.24	0.6	97.71%	251
Total	7033	3481.33	0.49	98.50%	377

In addition, 377 (5.36%) of the 7033 mapped SNPs showed segregation distortion (*P* < 0.05). The At genome included 126 (33.42%) and the Dt genome 251 (66.58%, Table 1). There was no distorted marker on chromosomes A07, A08, D03, D04, D07, D08, D11 and D12. Chromosome D06 had the largest number of distorted loci (82) (Fig. 1).

### QTL mapping of seed size, oil and protein content

Based on the high-density genetic map and genotype and trait data, a total of 58 QTL, including 12 for HSW, eight for HKW, six for TKL, six for TKW, 13 for KOC and 13 for KPC, were identified (Table 2; Fig. 1). These QTL explained 10.5–56.9% of the total phenotypic variance with LOD values ranging from 2.0 to 14.5. Among these QTL, 31 were on the At subgenome and 29 on the Dt (Table 2; Fig. 1). Twenty-two QTL had positive additive effects derived from Yumian 1, the others deriving from M11 (Table 2).

**Table 2:** QTL for cottonseed traits identified across three environments

Trait^a^	QTL	Env^b^	Flanking markers	Location	Nearest locus	LOD	Additive^c^	PVE^d^(%)
HSW	qHSWA01.1	2016CQ	Marker1095Marker1475	83.385	Marker1766	3.34	0.52	17.1
		2017AY	Marker1050Marker1444	80.444	Marker1682	3.07	0.45	15.2
	qHSWA01.2	2016CQ	Marker2119Marker2188	105.852	Marker2140	2.33	0.44	12.3
		2017AY	Marker2060Marker2271	108.794	Marker2270	2.76	0.43	13.7
	qHSWA09.1	2016CQ	Marker11851Marker11864	117.677	Marker11859	2.18	0.43	11.5
	qHSWA11.1	2016CQ	Marker13572Marker13721	59.099	Marker13615	3.87	0.60	19.5
		2016HN	Marker13572Marker13746	75.349	Marker13740	2.68	0.38	14
		2017AY	Marker13720Marker13746	75.349	Marker13740	2.65	0.44	13.2
	qHSWD02.1	2017AY	Marker18193Marker18204	40.414	Marker18200	2.07	0.38	10.5
	qHSWD02.2	2016CQ	Marker18224Marker18299	51.754	Marker18289	2.03	0.41	10.8
	qHSWD03.1	2016CQ	Marker18918Marker18934	107.899	Marker18928	9.22	0.83	40.4
		2016HN	Marker18918Marker18934	106.722	Marker18921	9.37	0.66	40.9
		2017AY	Marker18918Marker18940	107.899	Marker18928	9.01	0.76	38.3
	qHSWD05.1	2017AY	Marker19701Marker19762	119.944	Marker19721	2.48	0.56	12.4
	qHSWD05.2	2016CQ	Marker20000Marker20073	217.105	Marker20039	3.36	0.61	17.2
	qHSWD09.1	2016HN	Marker22228Marker22277	17.959	Marker22254	2.27	0.34	12
		2017AY	Marker22228Marker22277	18.555	Marker22255	3.75	0.50	18.2
	qHSWD09.2	2017HN	Marker22575Marker22599	116.855	Marker22592	2.23	0.35	11.8
		2017AY	Marker22575Marker22599	114.931	Marker22590	3.81	0.51	18.5
	qHSWD13.1	2017HN	Marker25633Marker25644	24.158	Marker25641	2.54	0.36	13.3
		2017AY	Marker25633Marker25662	24.158	Marker25641	4.07	0.52	19.6
HKW	qHKWA01.1	2016CQ	Marker1095Marker1248	83.973	Marker1779	3.14	0.32	17.6
		2016HN	Marker1095Marker1796	82.797	Marker1750	2.44	0.28	12.9
		2017AY	Marker1095Marker1796	83.973	Marker1777	3.51	0.39	18.3
	qHKWA01.2	2016CQ	Marker2060Marker2271	107.029	Marker2195	2.77	0.30	15.6
		2016HN	Marker2119Marker1822	107.029	Marker2195	2.32	0.28	12.4
		2017AY	Marker2060Marker2271	108.794	Marker2270	2.89	0.36	15.3
	qHKWA11.1	2016CQ	Marker13724Marker13746	75.349	Marker13740	2.47	0.30	14.1
		2016HN	Marker13572Marker13746	75.349	Marker13740	2.94	0.32	15.4
		2017AY	Marker13721Marker13746	75.349	Marker13740	2.4	0.34	12.9
	qHKWD01.1	2016HN	Marker17803Marker17824	69.664	Marker17810	2.05	0.27	11
	qHKWD01.2	2016CQ	Marker18034Marker18045	106.31	Marker18037	2.15	0.27	12.4
	qHKWD03..1	2016CQ	Marker18922Marker18940	107.899	Marker18928	14.15	0.61	58.1
		2016HN	Marker18918Marker18934	107.899	Marker18928	15.25	0.62	58
		2017AY	Marker18922Marker18940	112.14	Marker18935	10.64	0.65	45.8
	qHKWD09..1	2017AY	Marker22228Marker22290	18.555	Marker22255	3.83	0.41	19.8
	qHKWD13..1	2016CQ	Marker25627Marker25644	24.158	Marker25641	2.52	0.29	14.3
		2017AY	Marker25627Marker25644	24.158	Marker25641	3.15	0.38	16.6
TKL	qTKLA05.1	2016CQ	Marker6368Marker6440	49.956	Marker6433	4.04	1.27	20.8
		2016HN	Marker6391Marker6437	49.956	Marker6433	2.18	1.00	11.5
		2017AY	Marker6368Marker6437	45.089	Marker6388	3.82	1.45	19.1
	qTKLA11.1	2016CQ	Marker12954Marker13001	29.286	Marker12983	2.3	0.99	12.4
	qTKLA12.1	2016CQ	Marker13902Marker13921	6.443	Marker13906	2.5	1.05	13.4
		2016HN	Marker13904Marker13922	6.443	Marker13906	2.11	1.01	11.2
	qTKLD02.1	2016CQ	Marker18466Marker18541	72.256	Marker18639	3.83	1.25	19.8
		2016HN	Marker18541Marker18658	83.832	Marker18645	2.73	1.16	14.2
		2017AY	Marker18441Marker18643	72.256	Marker18639	3.22	1.35	16.3
	qTKLD08.1	2016HN	Marker21909Marker21965	49.622	Marker21942	2.24	1.02	11.8
	qTKLD13.1	2016CQ	Marker25619Marker25633	18.006	Marker25627	2.29	0.98	12.4
		2017AY	Marker25619Marker25633	17.389	Marker25620	2.53	1.22	13.1
TKW	qTKWA01.1	2016CQ	Marker1095Marker1796	80.444	Marker1683	2.58	0.76	14
		2016HN	Marker1651Marker1785	82.797	Marker1761	2.26	0.89	11.9
		2017AY	Marker1581Marker1599	77.502	Marker1590	2.1	0.72	11
	qTKWA08.1	2017AY	Marker10504Marker10552	69.174	Marker10502	2.05	0.72	10.7
	qTKWA11.1	2016CQ	Marker13572Marker13746	55.26	Marker13613	3.03	0.88	16.2
		2016HN	Marker13604Marker13722	57.005	Marker13614	2.56	1.01	13.4
		2017AY	Marker13572Marker13718	55.26	Marker13613	2.29	0.80	11.9
	qTKWD01.1	2017AY	Marker18034Marker18037	103.839	Marker18038	2.14	0.74	11.2
	qTKWD03.1	2016CQ	Marker18922Marker18940	107.899	Marker18928	14.86	1.63	58
		2016HN	Marker18918Marker18934	107.899	Marker18928	12.88	1.93	51.5
		2017AY	Marker18933Marker18978	112.14	Marker18935	9	1.43	39.3
	qTKWD09.1	2017AY	Marker22575Marker22599	114.931	Marker22588	2.85	0.85	14.6
KOC	qKOCA01.1	2016CQ	Marker990Marker1248	82.797	Marker1750	2.83	1.13	14.9
		2017AY	Marker1734Marker1798	83.973	Marker1774	2.43	1.07	12.2
	qKOCA01.2	2016CQ	Marker2014Marker2271	105.852	Marker2138	3.49	1.25	18
	qKOCA03.1	2017AY	Marker4951Marker5025	44.143	Marker4961	2.3	1.04	11.6
	qKOCA08.1	2016CQ	Marker10503Marker10582	72.718	Marker10567	2.69	1.11	14.2
	qKOCA09.1	2016HN	Marker10981Marker11001	37.502	Marker10991	3.69	1.24	18.7
	qKOCA11.1	2017AY	Marker13724Marker13746	72.492	Marker13726	2.07	1.04	10.5
	qKOCA13.1	2017AY	Marker16773Marker16984	136.821	Marker16970	2.4	1.07	12.1
	qKOCA13.2	2016CQ	Marker17148Marker17212	164.157	Marker17150	3.32	1.22	17.2
	qKOCD02.1	2016CQ	Marker18421Marker18439	62.907	Marker18431	3.2	1.20	16.6
	qKOCD03.1	2016CQ	Marker18933Marker18940	112.14	Marker18935	11.48	2.13	47.9
		2016HN	Marker18933Marker18940	112.14	Marker18935	3.54	1.26	18
		2017AY	Marker18918Marker18978	135.298	Marker18977	4.84	1.54	22.8
	qKOCD05.1	2017AY	Marker19712Marker19766	125.411	Marker19762	2.24	1.40	11.3
	qKOCD09.1	2016CQ	Marker22592Marker22602	120.918	Marker22600	2.02	0.99	10.8
		2017AY	Marker22600Marker22609	128.058	Marker22603	2.25	1.07	11.4
KPC	qKPCA01.1	2016CQ	Marker1050Marker1599	75.135	Marker1105	2.87	0.61	15.1
	qKPCA01.2	2017AY	Marker1766Marker1796	83.973	Marker1777	2.1	1.23	10.7
	qKPCA01.3	2016CQ	Marker2119Marker2271	105.852	Marker2140	3.95	0.70	20.1
	qKPCA08.1	2016CQ	Marker10273Marker10297	47.49	Marker10290	2.16	0.54	11.6
	qKPCA08.2	2016CQ	Marker10511Marker10600	72.13	Marker10561	2.75	0.59	14.5
	qKPCA09.1	2016HN	Marker10981Marker11001	37.502	Marker10991	2.88	1.51	14.9
	qKPCA11.1	2017AY	Marker13724Marker13740	72.492	Marker13726	2.12	1.29	10.7
	qKPCA13.1	2017AY	Marker17035Marker17090	151.357	Marker17079	2.12	1.25	10.7
	qKPCD02.1	2016CQ	Marker18429Marker18433	62.907	Marker18431	4.82	0.77	24
	qKPCD02.2	2016HN	Marker18645Marker18658	84.427	Marker18646	2.05	1.32	10.9
	qKPCD03.1	2016CQ	Marker18922Marker18940	112.14	Marker18935	8.81	1.03	39.4
		2016HN	Marker18933Marker18940	112.14	Marker18935	11.78	2.81	48.4
		2017AY	Marker18933Marker18940	112.14	Marker18935	9.91	2.52	41.2
	qKPCD09.1	2017AY	Marker22586Marker22599	116.855	Marker22592	2.53	1.39	12.7

^a^*HSW* hundred seed weight, *HKW* hundred kernel weight, *TKL* ten kernel length, *TKW* ten kernel width, *KOC* kernel oil content, *KPC* kernel protein content

^b^2016CQ, 2016 at Chongqing; 2016HN, 2016 at Hainan; 2017AY, 2017 at Anyang

^c^Positive additive effects indicated that Yumian 1 alleles increased the phenotypic value, negative additive effects suggested that M11 alleles increased the phenotypic value

^d^phenotypic variance explained

For HSW, 12 QTL were detected on eight chromosomes with LOD scores ranging from 2.03 to 9.08 and PVE ranging from 10.5 to 40% (Table 2; Fig. 1). The favorable alleles of eight QTL (qHSW-A09.1, qHSW-A11.1, qHSW-D02.1, qHSW-D02.2, qHSW-D03.1, qHSW-D03.2, qHSW-D05.2, qHSW-D09.2 and qHSW-D13.1) came from M11, and four (qHSW-A01.1, qHSW-A01.2, qHSW-D05.1 and qHSW-D09.1) came from Yumian1. Two QTL (qHSW-A11.1 and qHSW-D03.1) were detected across three environments and five (qHSW-A01.1, qHSW-A01.2, qHSW-D09.1, qHSW-D09.2 and qHSW-D13.1) across two environments.

Eight QTL for HKW were detected on six chromosomes, with LOD scores ranging from 2.05 to 13.47 (Table 2; Fig. 1). Among these QTL, five favorable QTL alleles increasing hundred kernel weight came from M11, whereas three originated from Yumian 1. Four QTL (qHKW-A01.1, qHKW-A01.2, qHKW-A11.1 and qHKW-D03.1) were detected across three environments, with PVE values of 18.3, 15.6, 15.4 and 53.5%, respectively.

Six QTL for ten kernel length were identified on chromosomes A05, A11, A12, D02, D08 and D13. The PVE of these QTL ranged from 11.2 to 20.8%. Among these QTL, two favorable alleles were contributed by Yumian 1 and the rest came from M11. However, only two QTL (qTKL-A05.1 and qTKL-D02.1) were identified in three environments.

Six QTL for TKW were detected on six chromosomes (Table 2; Fig. 1), with two on D03. The PVE values for these QTL ranged from 10.7 to 56.9%. Favorable alleles for four QTL (qTKW-A11.1, qTKW-D01.1, qTKW-D03.1 and qTKW-D09.1) derived from M11, while two (qTKW-A01.1 and qTKW-A08.1) were from Yumian1. Three QTL (qTKW-A01.1, qTKW-A11.1 and qTKW-D03.1) were detected in three environments.

Thirty QTL were detected for KOC on ten chromosomes, with PVE ranging from 10.5 to 48.2% and LOD scores ranging from 2.0 to 11.6 (Table 2; Fig. 1). Among them, favorable alleles for nine QTL (qKOC-A03.1, qKOC-A09.1, qKOC-A11.1, qKOC-A13.1, qKOC-A13.2, qKOC-D02.1, qKOC-D03.1, qKOC-D05.1 and qKOC-D09.1) were contributed by M11, and others (qKOC-A01.1, qKOC-A01.2, qKOC-A01.3 and qKOC-A08.1) came from Yumian1. Two QTL (qKOC-A01.2 and qKOC-D09.1) and one QTL (qKOC-D03.1) were identified across two and three environments, respectively.

Thirty QTL for KPC were mapped on eight chromosomes, explaining 10.7–49.1% of the phenotypic variance (Table 2; Fig. 1). Chromosomes A01, A08 and D02 contained four, two and two QTL on different regions, respectively. Among these QTL, seven favorable alleles increasing trait value came from Yumian 1, whereas the rest were from M11. One QTL (qKPC-D03.1) was detected in three environments.

### QTL hotpots/cluster

In this study, we found seven QTL clusters distributed on 6 chromosomes, including three on the At subgenome and four on the Dt subgenome (Table 3). Every QTL cluster possessed at least three QTL for different traits. A01-cluster-1 had the highest number of QTL (7 QTL for qHKW, qHSW, qTKW, qKOC and qKPC). D03-cluster-1, A11-cluster-1 and A01-cluster-1 contained five, three and two stable QTL, respectively. These QTL clusters could be priorities for further application (Table 3).

**Table 3:** QTL clusters for cottonseed traits identified across in the Yumian 1 × M11 RIL population

QTLs^a^	Flanking markers
A01-cluster-1	
qHKW-A01.1	Marker990-Marker1444
qHSW-A01.1	
qTKW-A01.1	
qKOC-A01.1	
qKOC-A01.2	
qKPC-A01.1	
qKPC-A01.2	
A01-cluster-2	
qHKW-A01.2	Marker2014-Marker2271
qHSW-A01.2	
qKOC-A01.3	
qKPC-A01.3	
A11-cluster-1	
qHKW-A11.1	Marker13572-Marker13746
qHSW-A11.1	
qTKW-A11.1	
qKOC-A11.1	
qKPC-A11.1	
D02-cluster-1	
qTKL-D02.1	Marker18441-Marker18658
qKOC-D02.1	
qKPC-D02.1	
D03-cluster-1	
qHKW-D03..1	Marker18918-Marker18978
qHSW-D03.1	
qTKW-D03.1	
qKOC-D03.1	
qKPC-D03.1	
D09-cluster-1	
qHSW-D09.2	Marker22575-Marker22602
qTKW-D09.1	
qKOC-D09.1	
D13cluster-1	
qHKW-D13..1	Marker25619-Marker25662
qHSW-D13.1	
qTKL-D13.1	

^a^*HSW* hundred seed weight, *HKW* hundred kernel weight, *TKL* ten kernel length, *TKW* ten kernel width, *KOC* kernel oil content, *KPC* kernel protein content

## Discussion

### Correlation between seed size, oil and protein content

After measuring seed size, oil and protein content, we analyzed the correlation among these traits. Beyond the significant correlation between seed weight (HSW, same as seed index, and HKW) and oil and protein content (KOC and KPC), as described by Pahlavani et al. [4], we found that kernel shape (TKL and TKW) was significantly correlated with seed weight (HSW and HKW), oil and protein content (KOC and KPC). TKW was more closely correlated with KOC and KPC than TKL (Additional file 2: Table S2). Approximately 80% of the dry weight of the cottonseed kernel consists of storage lipid and protein, and cotyledon tissue accounts for 60% of the cottonseed kernel [26]. Due to the physical shape of cotyledons, their influence on kernel width may be larger than kernel length. In addition, there is rapid accumulation of oil and storage protein in the embryo maturation stage over 25–45 DPA with increased size and weight of the cotyledons [27, 28]. This growth trajectory may be the reason that TKW was more significantly correlated with KOC and KPC than TKL and further study is needed to understand the correlation between kernel shape and other traits.

### The direction of favorable QTL alleles

The favorable alleles for a trait do not necessarily come from the more favorable parent. For instance, Liu et al. [14] identified 14 QTL for seed index, with five favorable alleles coming from Yumian1 and the remainder from CCRI35. Zhang et al. [22] detected 16 stable QTL for boll weight, including 8 whose favorable alleles came from the maternal parent and 8 from the paternal parent. Among 60 QTL detected in the present study, 22 favorable alleles came from Yumian 1 with the rest from M11 (Table 2). This result, combined with previous reports, indicated that both the superior and inferior parent could contribute QTL alleles that increase the trait value, contributing to transgressive segregation in progeny populations.

### Stable and common QTL

Stable and major QTL for yield and quality are important to molecular breeding. It is well known that quantitative traits are controlled by multiple genes and affected by environment [14]. In the present study, variance analysis also suggested a significant influence of environment on the development of cottonseed. Hence, this study considered QTL identified in all test environments as stable QTL. Thirteen stable QTL were detected, most of which were within QTL clusters/hotpots (Table 2, Table 3). These stable QTL deserve priority for further research, including fine mapping, candidate gene identification and molecular mechanism analysis of cottonseed development. Moreover, these stable QTL have the potential to improve cottonseed quality through MAS.

Until now, QTL or SNP associated with seed index have been identified by traditional QTL mapping methods or GWAS [8, 17, 29]. We compared the stable QTL detected in this study with QTL identified in previous studies through the physical position of the nearest marker(s). Two stable QTL had been previously reported, while 11 (qHKW-A01.1, qHKW-A01.2, qHKW-A11.1, qHKW-D03.1, qTKL-A05.1, qTKL-D02.1, qTKW-A01.1, qTKW-A11.1, qTKW-D03.1, qKOC-D03.1 and qKPC-D03.1) were newly found. Identifying candidate genes controlling these new QTL for kernel length and kernel width will accelerate research into the mechanism of cottonseed growth. These common QTL and novel stable QTL would be priorities for MAS to improve cottonseed quality by transferring favorable alleles into cotton cultivars.

## Methods

### Population construction

A RIL population including 180 lines was developed from a cross between Yumian 1, a high fiber quality cultivar, was bred through a multiple-line intermating program [30]; and M11, a high oil line provided by Dr. Du from Cotton Research Institute. The parents were crossed at Southwest University, Chongqing, China, in the summer of 2010. The F_1_ seeds were planted in Hainan, China, in the winter of the same year. In the summer of 2011, 180 F_2_ plants were randomly selected. Since then, single-seed descent was executed from F_2:3_ to F_2:8_. The RIL population was formed in the summer of 2015. All RIL lines along with two parents were planted in Chongqing, China, in the summer of 2016, Hainan, China, in the winter of 2016 and Anyang, China, in the summer of 2017, respectively.

### Phenotypic data analysis

All naturally opened bolls were hand-harvested. After ginning and drying, one hundred seeds were selected randomly and weighed to determine seed index (HSW, g). Subsequently, the cottonseed kernels were firstly used to measure hundred kernel weight (HKW, g), ten kernel length (TKL, mm) and ten kernel width (TKW, mm) after hulling. Then, the kernels were ground into powder to detect oil (KOC) and protein (KPC) content by Fourier Transform Infrared Spectrometer (NIRFlex® N-500). The frequency distribution and correlation coefficients among these traits were analyzed by SPSS version 20.0 (SPSS, Chicago, IL, USA), and the phenotypic trends and the relevance of these traits were illustrated intuitively in box plot drawings by Plotly 2.0 (https://plot.ly).

### DNA preparation, SLAF-library construction, and high throughput sequencing

Total genomic DNA was extracted from fresh young leaves of two parents and 86 RILs according to a modified CTAB method by Zhang et al. [31]. The SLAF-seq strategy for library construction was according to Shen et al. [24] with some modifications. The cotton reference genome used in this study was released by Zhang et al. [16]. A pilot experiment was carried out to determine the enzymes selected for library construction and the size of the restriction fragments for SLAFs. Clean DNA was digested into fragments with the specific enzyme combinations RsaI+HaeIII (NEB, Ipswich, MA, USA.). After a series of treatments to these restriction fragments, high-throughput sequencing was performed using an Illumina HiSeqTM-2500 (Illumina, Inc., San Diego, CA, USA) at the Biomarker Technologies Corporation in Beijing. Subsequently, examination was performed to evaluate the result of sequencing.

### Sequencing data grouping and genotyping

SLAF identification and genotyping was based on procedures described by Sun et al. [32] and Shen et al. [24]. Initially, low-quality reads (quality score < 20e) were filtered out and the remaining reads were arranged for the progenies according to the duplex barcode sequences. Then, 5 bp terminal sites were trimmed, to yield high quality reads. The *G. hirsutum* reference genome was retrieved from Phytozome (https://phytozome.jgi.doe.gov/Ghirsutum_er). Clean reads were mapped to the reference genome using Burrows-Wheeler-Aligner (BWA) software [33]. Sequences were defined as one SLAF marker if they mapped on the same position with over 95% identity [16]. Subsequently, GATK software and Samtools/bcftools were used to detect SNPs between the parents [34–36]. SNPs of low quality were filtered out, based on the following criteria: a) minimum read depth less than 10; b) average base quality less than 30; c) SNPs in each RIL anchored on different position; and d) SNPs in RILs with more than 40% missing data [24].

### Map construction and segregation distortion analysis

HighMap was used to order the SLAF markers, correct genotyping errors within the chromosomes and calculate the genetic distance between adjacent marker. Besides, SMOOTH was applied to correct errors based on the parental contribution of genotypes, and a k-nearest neighbor algorithm was used to impute missing genotypes as described by Zhang et al. [22]. Chi-squared tests were employed to test loci for deviation from the 1:1 expected segregation ratio (*p* < 0.05).

### QTL analysis

The QTL influencing cottonseed size, oil and protein content were identified by MapQTL 6.0 [37], using multiple QTL mapping. A threshold of log of odds ratio (LOD) ≥ 2.0 was used to declare suggestive QTL as suggested by Lander and Kruglyak [38]. Positive additive effects indicated favorable alleles derived from M11, while negative additive effects indicated favorable alleles from Yumian 1. The QTL nomenclature was designated as: q + trait abbreviation + chromosome number + QTL number. QTL identified in three environments were considered stable.

## Additional files

Additional file 1: **Table S1.** Variation of cottonseed traits for the RIL population in three environments. (XLSX 12 kb)

Additional file 2: **Table S2.** Correlation analysis among fiber quality traits across three environments. (XLSX 10 kb)

Additional file 3: **Table S3.** Analysis of variance (ANOVA) for cottonseed traits across three environments for the Yumian 1 × M11 RIL population. (XLSX 10 kb)

Additional file 4: **Table S4.** Characteristics of SLAFs and SNPs. (XLSX 9 kb)

Additional file 5: **Figure S1.** Phenotypic distribution of cottonseed quality traits in the Yumian 1 × M11 RIL population. (PNG 54 kb)

Additional file 6: **Figure S2.** SLAF marker distribution on the *Gossypium hirsutum* genome. (TIF 908 kb)

Additional file 7: **Supplement S1.** Genetic maps of the Yumian 1 × M11 RIL population. (PDF 140 kb)

## Data Availability

Sequencing data related to this study has been uploaded to NCBI SRA database, which can be accessed through series of SRA numbers PRJNA532305.

## Abbreviations

Chr: Chromosome
GBS: Genotyping by sequencing
HKW: Hundred kernel weight
HSW: Hundred seed weight
KOC: Kernel oil content
KPC: Kernel protein content
LOD: Log of odds ratio
MAS: Marker assisted selection
NGS: Next generation sequencing
PVE: Phenotypic variance explained
QTL: Quantitative trait locus/loci
RAD: Restriction-site associated DNA
RIL: Recombinant inbred line
SLAF-seq: Specific locus amplified fragment sequencing
SNP: Single nucleotide polymorphism
SSR: Simple sequence repeat(s)
TKL: ten kernel length
TKW: ten kernel width

## References

[1] Zhang K, Zhang J, Ma J, Tang S, Liu D, Teng Z, Liu D, Zhang Z (2011). Genetic mapping and quantitative trait locus analysis of fiber quality traits using a three-parent composite population in upland cotton (*Gossypium hirsutum* L.). Mol Breed.

[2] Sawan ZM, Elfarra AA, Ellatif SA (1988). Cottonseed, protein and oil yields, and oil properties as affected by nitrogen and phosphorus fertilization and growth-regulators. J Agron Crop Sci.

[3] Ahmad S, Anwar F, Hussain AI, Ashraf M, Awan AR (2007). Does soil salinity affect yield and composition of cottonseed oil?. J Am Oil Chem Soc.

[4] Pahlavani M., Miri A., Kazemi G. (2009). Response of oil and protein content to seed size in cotton(Gossypium hirsutum L., cv. Sahel). Plant Breeding and Seed Science.

[5] Kothari N, Campbell BT, Dever JK, Hinze LL (2016). Combining ability and performance of cotton germplasm with diverse seed oil content. Crop Sci.

[6] Hanny BW, Meredith WR, Bailey JC, Harvey AJ (1978). Genetic relationships among chemical constituents in seeds, flower buds, terminals, and mature leaves of cotton. Crop Sci.

[7] Yu JW, Yu SX, Fan SL, Song MZ, Zhai HH, Li XL, Zhang JF (2012). Mapping quantitative trait loci for cottonseed oil, protein and gossypol content in a *Gossypium hirsutum* x *Gossypium barbadense* backcross inbred line population. Euphytica..

[8] Said JI, Knapka JA, Song MZ, Zhang JF (2015). Cotton QTLdb: a cotton QTL database for QTL analysis, visualization, and comparison between *Gossypium hirsutum* and *G. hirsutum* x *G. barbadense* populations. Mol Gen Genomics.

[9] Song Xian-Liang, Zhang Tian-Zhen (2007). Identification of quantitative trait loci controlling seed physical and nutrient traits in cotton. Seed Science Research.

[10] An C, Jenkins JN, Wu J, Guo Y, McCarty JC (2009). Use of fiber and fuzz mutants to detect QTL for yield components, seed, and fiber traits of upland cotton. Euphytica..

[11] Alfred Q, Liu HY, Xu HM, Li JR, Wu JG, Zhu SJ, Shi CH (2012). Mapping of quantitative trait loci for oil content in cottonseed kernel. J Genet.

[12] Liu D, Liu F, Shan X, Zhang J, Tang S, Fang X, Liu X, Wang W, Tan Z, Teng Z (2015). Construction of a high-density genetic map and lint percentage and cottonseed nutrient trait QTL identification in upland cotton (*Gossypium hirsutum* L.). molecular genetics and genomics. Mol Gen Genomics.

[13] Shang LG, Abduweli A, Wang YM, Hua JP (2016). Genetic analysis and QTL mapping of oil content and seed index using two recombinant inbred lines and two backcross populations in upland cotton. Plant Breed.

[14] Liu XY, Teng ZH, Wang JX, Wu TT, Zhang ZQ, Deng XP, Fang XM, Tan ZY, Ali I, Liu DX (2017). Enriching an intraspecific genetic map and identifying QTL for fiber quality and yield component traits across multiple environments in upland cotton (*Gossypium hirsutum* L.). Mol Gen Genomics.

[15] Paterson AH, Wendel JF, Gundlach H, Guo H, Jenkins J, Jin DC, Llewellyn D, Showmaker KC, Shu SQ, Udall J (2012). Repeated polyploidization of *Gossypium* genomes and the evolution of spinnable cotton fibres. Nature..

[16] Zhang TZ, Hu Y, Jiang WK, Fang L, Guan XY, Chen JD, Zhang JB, Saski CA, Scheffler BE, Stelly DM (2015). Sequencing of allotetraploid cotton (*Gossypium hirsutum* L. acc. TM-1) provides a resource for fiber improvement. Nat Biotechnol.

[17] Du XM, Huang G, He SP, Yang ZE, Sun GF, Ma XF, Li N, Zhang XY, Sun JL, Liu M (2018). Resequencing of 243 diploid cotton accessions based on an updated a genome identifies the genetic basis of key agronomic traits. Nat Genet.

[18] Wang M, Tu L, Yuan D, Zhu SC, Li J, Liu F, Pei L, Wang P, Zhao G, et al. Reference genome sequences of two cultivated allotetraploid cottons, *Gossypium hirsutum* and *Gossypium barbadense*. Nat Genet. 2018. 10.1038/s41588-018-0282-x 30510239

[19] Wei Qingzhen, Wang Yunzhu, Qin Xiaodong, Zhang Yunxia, Zhang Zhentao, Wang Jing, Li Ji, Lou Qunfeng, Chen Jinfeng (2014). An SNP-based saturated genetic map and QTL analysis of fruit-related traits in cucumber using specific-length amplified fragment (SLAF) sequencing. BMC Genomics.

[20] Wang S, Chen JD, Zhang WP, Hu Y, Chang LJ, Fang L, Wang Q, Lv FN, Wu HT, Si ZF, et al. Sequence-based ultra-dense genetic and physical maps reveal structural variations of allopolyploid cotton genomes. Genome Biol. 2015;16. 10.1186/s13059-015-0678-1 PMC4469577 26003111

[21] Jia XY, Pang CY, Wei HL, Wang HT, Ma QF, Yang JL, Cheng SS, Su JJ, Fan SL, Song MZ, et al. High-density linkage map construction and QTL analysis for earliness-related traits in *Gossypium hirsutum* L. BMC Genomics. 2016;17. 10.1186/s12864-016-3269-y PMC5106845 27835938

[22] Ali I, Teng ZH, Bai YT, Yang Q, Hao YS, Hou J, Jia YB, Tian LX, Liu XY, Tan ZY, et al. A high density SLAF-SNP genetic map and QTL detection for fibre quality traits in *Gossypium hirsutum*. BMC Genomics. 2018;19. 10.1186/s12864-018-5294-5 PMC6282304 30522437

[23] Qi HK, Wang N, Qiao WQ, Xu QH, Zhou H, Shi JB, Yan GT, Huang Q. Construction of a high-density genetic map using genotyping by sequencing (GBS) for quantitative trait loci (QTL) analysis of three plant morphological traits in upland cotton (*Gossypium hirsutum* L.). Euphytica. 2017;213(4).

[24] Shen C, Jin X, Zhu D, Lin ZX. Uncovering SNP and indel variations of tetraploid cottons by SLAF-seq. BMC Genomics. 2017;18. 10.1186/s12864-017-3643-4 PMC5363057 28330454

[25] Zhang Z, Shang HH, Shi YZ, Huang L, Li JW, Ge Q, Gong JW, Liu AY, Chen TT, Wang D, et al. Construction of a high-density genetic map by specific locus amplified fragment sequencing (SLAF-seq) and its application to quantitative trait loci (QTL) analysis for boll weight in upland cotton (*Gossypium hirsutum*.). BMC Plant Biol. 2016;16:79. 10.1186/s12870-016-0741-4 PMC4827241 27067834

[26] McDaniel RG. Physiological and scanning electron microscopic evaluations of cottonseed quality. Phoenix,1979; Arizona: 40–42.

[27] Reeves RG, Beasley JO (1935). The development of the cotton embryo. J Agric Res.

[28] Forman M, Jensen WA (1965). Respiration and embryogenesis in cotton. Plant Physiol.

[29] Fang L, Wang Q, Hu Y, Jia YH, Chen JD, Liu BL, Zhang ZY, Guan XY, Chen SQ, Zhou BL (2017). Genomic analyses in cotton identify signatures of selection and loci associated with fiber quality and yield traits. Nat Genet.

[30] Zhang ZS, Hu MC, Zhang J, Liu DJ, Zheng J, Zhang K, Wang W, Wan Q (2009). Construction of a comprehensive PCR-based marker linkage map and QTL mapping for fiber quality traits in upland cotton (*Gossypium hirsutum* L.). Mol Breed.

[31] Zhang ZS, Xiao YH, Luo M, Li XB, Luo XY, Hou L, Li DM, Pei Y (2005). Construction of a genetic linkage map and QTL analysis of fiber-related traits in upland cotton (*Gossypium hirsutum* L.). Euphytica..

[32] Sun Xiaowen, Liu Dongyuan, Zhang Xiaofeng, Li Wenbin, Liu Hui, Hong Weiguo, Jiang Chuanbei, Guan Ning, Ma Chouxian, Zeng Huaping, Xu Chunhua, Song Jun, Huang Long, Wang Chunmei, Shi Junjie, Wang Rui, Zheng Xianhu, Lu Cuiyun, Wang Xiaowu, Zheng Hongkun (2013). SLAF-seq: An Efficient Method of Large-Scale De Novo SNP Discovery and Genotyping Using High-Throughput Sequencing. PLoS ONE.

[33] Li H, Durbin R (2009). Fast and accurate short read alignment with burrows-wheeler transform. Bioinformatics..

[34] DePristo MA, Banks E, Poplin R, Garimella KV, Maguire JR, Hartl C, Philippakis AA, del Angel G, Rivas MA, Hanna M (2011). A framework for variation discovery and genotyping using next-generation DNA sequencing data. Nat Genet.

[35] Li H, Handsaker B, Wysoker A, Fennell T, Ruan J, Homer N, Marth G, Abecasis G, Durbin R, Proc GPD (2009). The sequence alignment/map format and SAMtools. Bioinformatics..

[36] Li H (2011). A statistical framework for SNP calling, mutation discovery, association mapping and population genetical parameter estimation from sequencing data. Bioinformatics..

[37] Van Ooijen JW. MapQTL 6.0. Software for the Mapping of Quantitative Trait Loci in Experimental Populations. 2009; Wageningen: Kyazma, B.V.

[38] Lander E, Kruglyak L (1995). Genetic dissection of complex traits - guidelines for interpreting and reporting linkage results. Nat Genet.

