# Supplementary figures and images for: A high density SLAF-seq SNP genetic map and QTL for seed size, oil and protein content in upland cotton
Source: BMC Genomics. 2019 Jul 22;20:599. doi: 10.1186/s12864-019-5819-6 (PMC6647295; doi:10.1186/s12864-019-5819-6)

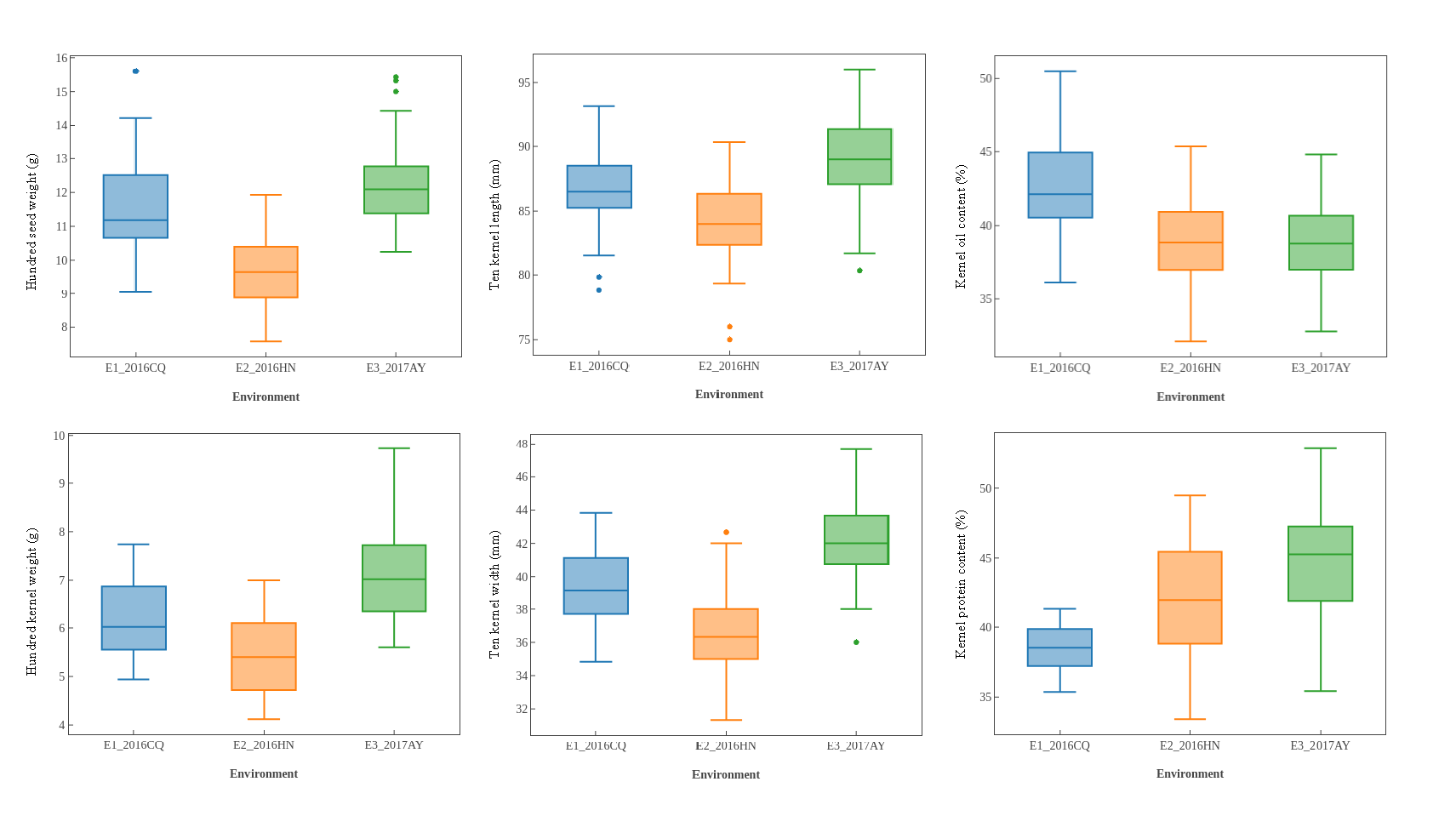

Supplement: Supplementary file 5 — Figure S1. Phenotypic distribution of cottonseed quality traits in the Yumian 1 × M11 RIL population. (PNG 54 kb) [file 12864_2019_5819_MOESM5_ESM.png]

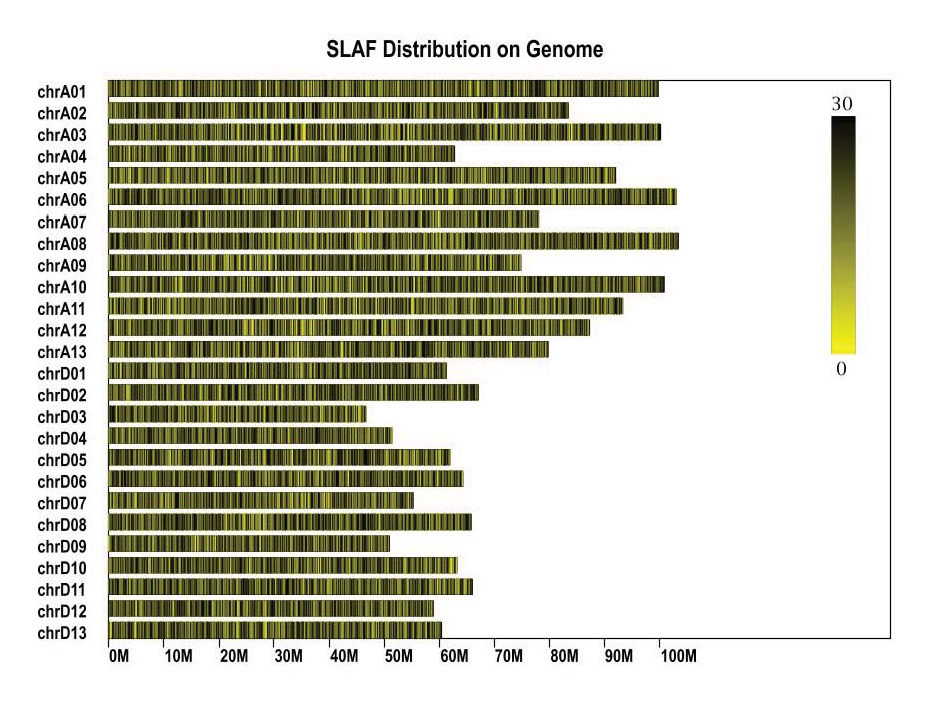

Supplement: Supplementary file 6 — Figure S2. SLAF marker distribution on the Gossypium hirsutum genome. (TIF 908 kb) [file 12864_2019_5819_MOESM6_ESM.tif]
